# Green Phellodendri Chinensis Cortex-based carbon dots for ameliorating imiquimod-induced psoriasis-like inflammation in mice

**DOI:** 10.1186/s12951-021-00847-y

**Published:** 2021-04-15

**Authors:** Meiling Zhang, Jinjun Cheng, Jie Hu, Juan Luo, Yue Zhang, Fang Lu, Hui Kong, Huihua Qu, Yan Zhao

**Affiliations:** 1 School of Traditional Chinese Medicine, Beijing University of Chinese Medicine, Beijing, 100029 China; 2 School of Life Sciences, Beijing University of Chinese Medicine, Beijing, 100029 China; 3 Center of Scientific Experiment, Beijing University of Chinese Medicine, Beijing, 100029 China

**Keywords:** Phellodendri Chinensis Cortex-based carbon dots, Psoriasis, Protective effect, Macrophage polarization, Imiquimod

## Abstract

**Background:**

Carbon dots (CDs) with multifaceted advantages have provided hope for development brand-new nanodrug for treating thorny diseases. This study developed a green and simple calcination method to prepare novel CDs as promising drug for psoriasis treatment. The as-prepared CDs using Phellodendri Chinensis Cortex (PCC) as sole precursor were characterized by a series of methods, mainly including electron microscopy, optical technology and X-ray photoelectron spectroscopy (XPS).

**Results:**

Results displayed that fluorescence (Quantum yield = 5.63%) and nontoxic PCC-based CDs (PCC-CDs) with abundant chemical groups exhibited solubility and tiny sizes at average of (1.93 ± 0.53) nm, which may be beneficial for its inherent biological activity. Moreover, by using the typical imiquimod (IMQ)-induced psoriasis-like skin mouse model, we firstly demonstrated the pronounced anti-psoriasis activity of as-prepared PCC-CDs on ameliorating the appearance, psoriasis area and severity index (PASI) scores as well as histopathological morphology of both back skin tissues and right ears in IMQ-induced mouse. Further potential mechanisms behind the anti-psoriasis activities may be related to suppress M1 polarization and relatively promote M2 polarization of macrophage both in vitro and in vivo.

**Conclusion:**

These results suggested that PCC-CDs have potential to be an anti-psoriasis candidate for clinical applications to treat psoriasis, which not only provided an evidence for further broadening the biological application of CDs, but also provided a potential hope for application nanodrugs to treat thorny diseases.

**Graphic Abstract:**

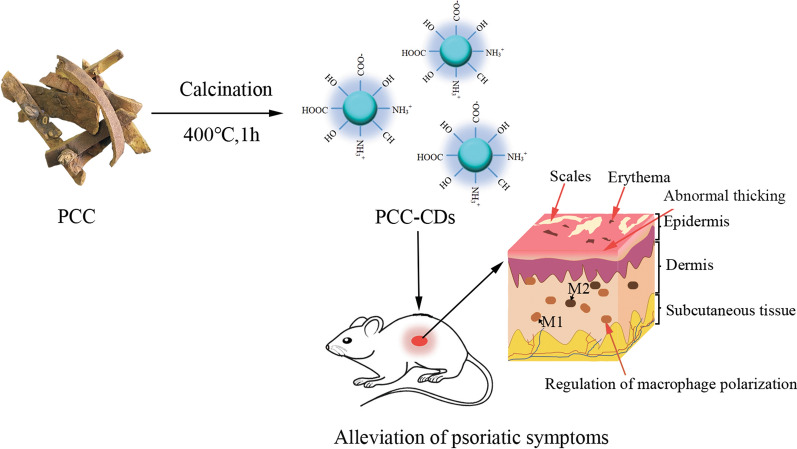

**Supplementary Information:**

The online version contains supplementary material available at 10.1186/s12951-021-00847-y.

## Background

Psoriasis, known as a common and current incurable chronic inflammatory dermatologic disorder, has affected approximately 2-3% of the global population [1]. The invaded skins were associated with a series of pathological features characterized by epidermal hyperproliferation, parakeratosis and inflammatory cells (such as macrophages, neutrophils, dendritic cells) infiltrates [2]. Increasing risk of other diseases consisting of metabolic syndrome, cardiovascular and arthritis diseases [3, 4] often occurs during the course of psoriasis, which caused a substantial decline on the patient’s quality of life. However, current temporary strategies including medicines (like glucocorticosteroids) , photo-therapies, and biological treatments [5] were not satisfied by suffers due to the severe side effects and financial burdens, making a safer and low-cost drug urgent to render this disease.

The gradually development of new nanomaterials-carbon dots (CDs) in the fields of biological applications have opened a promising avenue for a new generation of nanodrug for treatment multiple diseases due to their specific properties such as low or non-toxicity, tiny size, solubility and excellent biocompatibility [6–8]. Additionally, excessive evidences reported CDs derived different precursors [9–11] or preparing conditions (preparing temperature, time, etc) [12–14] showed different properties, mainly performed on the aspects of size, charge and chemical groups. These differences in properties have been demonstrated to be closely connected with the bioactivities of CDs, which further provided a rational explanation for the fact that CDs currently found possessed different activities including anti-inflammation [15], antioxidant [16], anticonception [17], anti-bacteria [18, 19] and hemostasis [20]. Based on this, the undiscovered and study-worthy potentials of CDs for controlling or treating other diseases such as psoriasis become the focus of current research, which deserved to be conducted.

**Fig 1.**
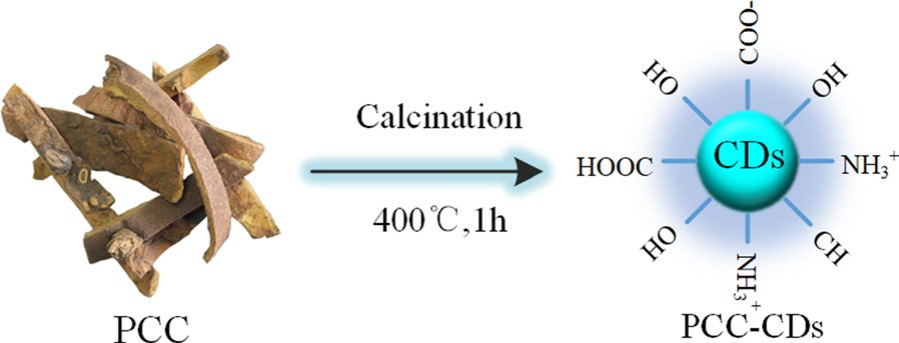
Illustration for as-prepared Phellodendri Chinensis Cortex-based carbon dots (PCC-CDs) by one-step calcination method

Herein, a novel CDs prepared by green and one-step calcination method (Fig. 1) using Phellodendri Chinensis Cortex (PCC) as sole carbon sources, and their positive effect on imiquimod (IMQ)-induced psoriasis-skin inflammation have been studied for the first time. This study further estimated M1/M2 related cytokines in serum and skin tissues and histopathological changes responsible for the observed effect. M1/M2 polarization model on RAW264.7 (mice peritoneal macrophages cell line) were further applied to verify the regulating of M1/M2 polarization of PCC-CDs in vitro. Moreover, the physicochemical properties of PCC-based CDs (PCC-CDs) including morphology, optical properties, functional groups were characterized, followed by the safety assessment via a cell counting kit-8 (CCK-8) assay.

## Results

### Characterization of PCC-CDs

**Fig. 2:**
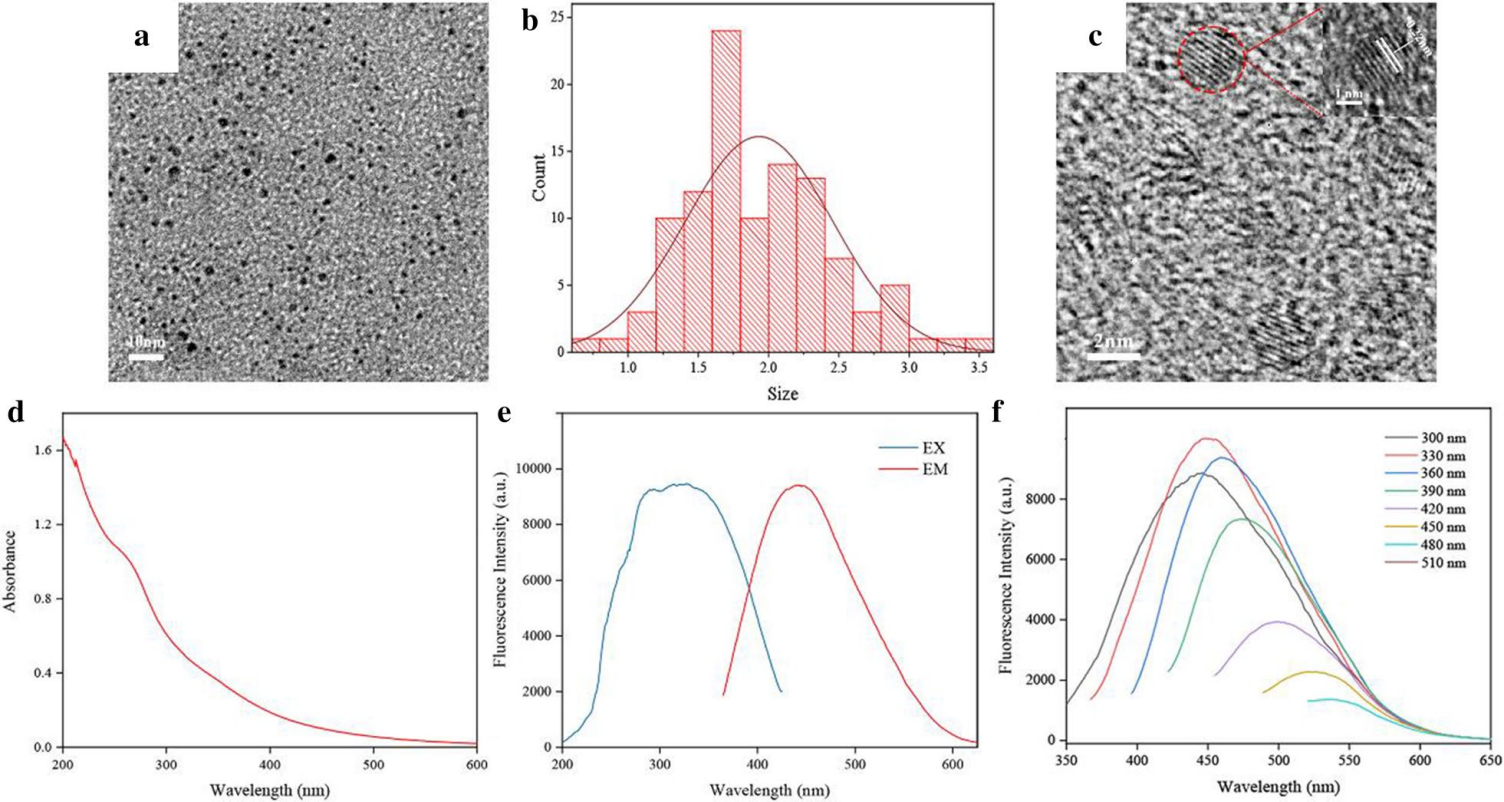
Morphological and optical characterizations of Phellodendri Chinensis Cortex-based carbon dots (PCC-CDs) prepared at 400 ℃. **a** Transmission electron microscopy (TEM) images. **b** Particle size distribution histogram. **c** High-resolution transmission electron microscopy (HRTEM). Inset: magnification figure. **d** Ultraviolet and visible spectrum, **e** Fluorescence spectra and **f** Fluorescence spectra of PCC-CDs with different excitation wavelengths

Transmission electron microscope (TEM) image (Fig. 2a) exhibited PCC-CDs prepared at 400 ℃ possessed monodispersed and quasi-spherical structure with size diameters ranging from 0.5 nm to 3.6 nm (Fig. 2b). Distinct lattices of 0.22 nm indicated the crystalline structure of PCC-CDs, which may be contributed to the existing of (102) crystal plane of graphite [21] (Fig. 2c).

The optical properties of PCC-CDs were shown on ultraviolet and visible (UV–Vis) spectra and fluorescence spectra. As illustrated as Fig.2d, broad absorption around 270 nm and 350 nm separately revealed the presence of π–π* and n–π* electronic transitions of PCC-CDs, which related to the different superficial states such as C=C, C=O, C=N and C–O on obtained CDs [22].In addition, the fluorescence CDs with quantum yield (QY) of 5.63% exhibited maximum emission and excitation wavelength at 445 nm and 330 nm, respectively (Fig. 2e). Figure 2f shows that the fluorescent peak of ZR-CDs was red-shifted with an increase in the excitation wavelength from 300 to 510 nm at 30 nm intervals, indicating that PCC-CDs possessed excitation-dependent emission properties.

**Fig 3.**
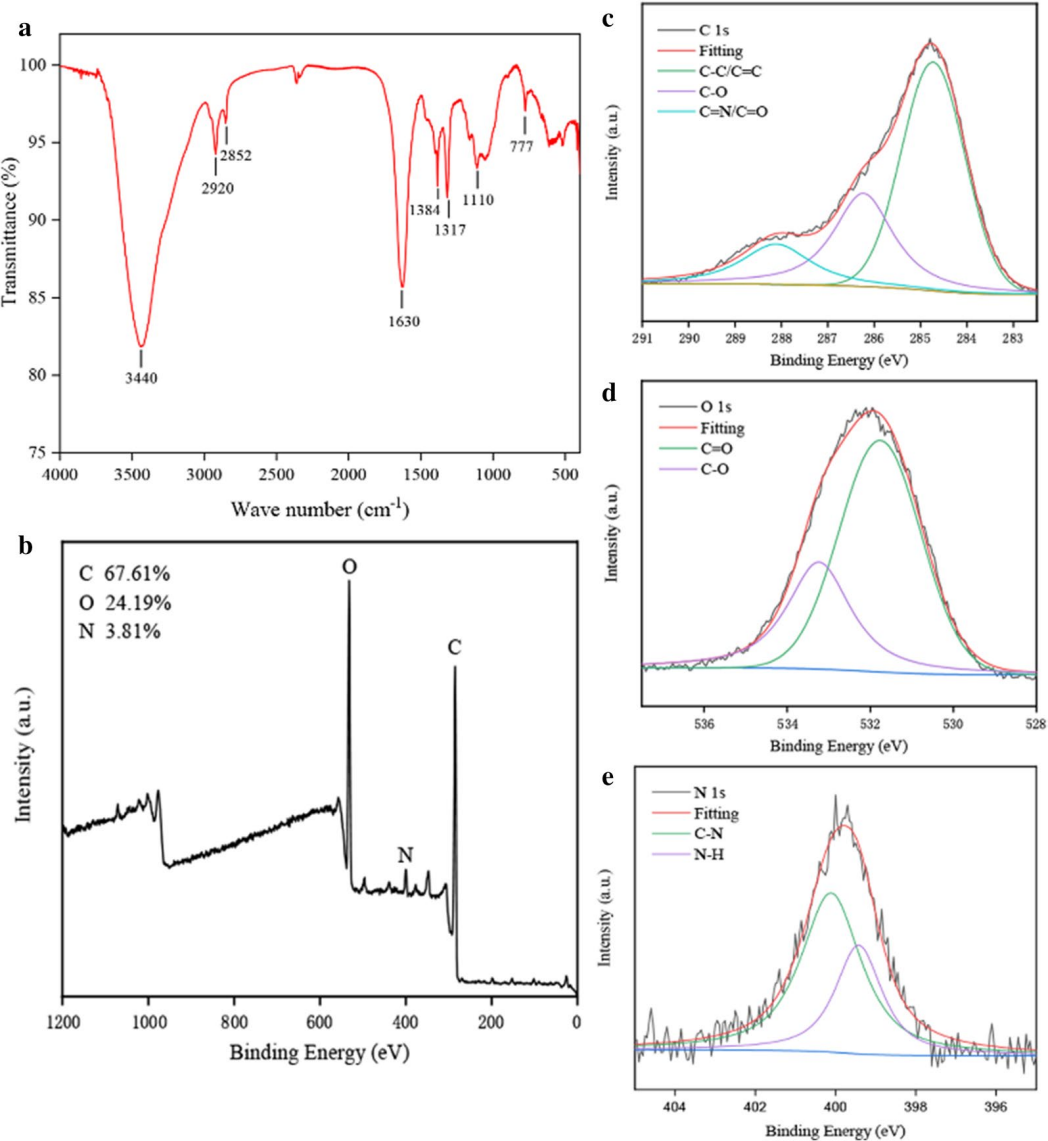
Functional groups and element analysis of Phellodendri Chinensis Cortex-based carbon dots (PCC-CDs). **a** Fourier transform infrared spectroscopy spectrum (FT-IR). X-ray photoelectron spectroscopy (XPS) spectrum including **b** full survey spectrum and **c** C 1s, **d** O 1s and **e** N 1s high-resolution survey spectrum

The surface functional groups and element composition of CDs derived from PCC were investigated with Fourier transform infrared spectroscopy spectrophotometer (FTIR) and X-ray photoelectron spectroscopy (XPS) techniques, as seen in Fig. 3. FTIR spectra (Fig. 3a) depicted that the broad absorption at 3440 cm^−1^ was attributed to stretching vibration of O/N–H bonds, while the peak at 2920 cm^−1^ and 2853 cm^−1^ belong to the presence of –CH_3_ and –CH_2_. Stretching vibration of C=O and amide groups were noted as 1630 cm^−1^, 1384 cm^−1^ and 1317 cm^−1^, respectively. Peak located at 1070 cm^−1^ suggested the existing of aromatic alkoxy bonds. The above-mentioned results indicated that the surface of CDs existed multi-functional groups such as hydroxyl, amino and carbonyl [23].

Furthermore, three predominant elements (C: 67.61%, O: 24.19%, N: 3.81%) could be observed in the XPS spectra of as-prepared PCC-CDs, as shown in Fig. 3b–e. The high-resolution C1s spectrum displayed distinct peaks at 284.7 eV, 286.2 eV and 288.5eV, which were assigned to the presence of C=C/C–C, C–O and C=O/C=N [24], respectively. Characteristic peaks located at 531.7 eV and 533.2 eV in high-resolution O1s spectrum were separately corresponded to C–O and C=O groups. Two peaks at around 399.4 and 400.1 eV existed in N1s spectra could be attributed to N–H and C–N bond, respectively [25]. The XPS spectra supported that various functional groups such as hydroxyl, amino and carbonyl were existed on the surface of PCC-CDs, which was consistent with the result of FTIR spectrum.

### Cytotoxicity assay

**Fig 4.**
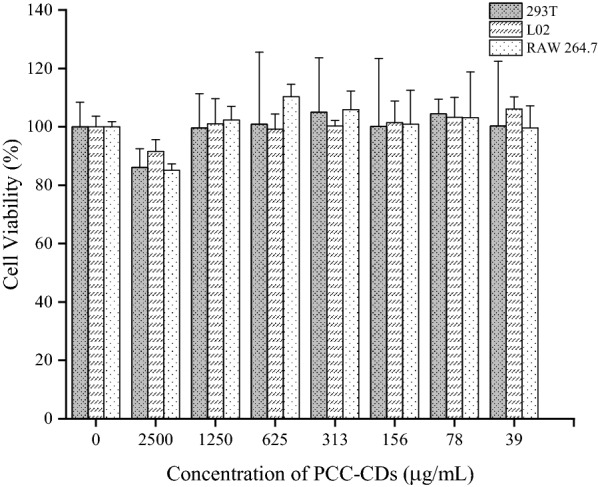
Cytotoxic effects of Phellodendri Chinensis Cortex-based carbon dots (PCC-CDs) at increasing concentrations on L02, 293T and RAW 264.7 cells via CCK-8 assay for 24 h

As potential drug materials possessed series of remarkable properties, CDs always triggered great concern about their biosafety. Fig. 4 showed gradually increasing PCC-CDs concentrations ranging from 39 μg/mL to 1250 μg/mL have almost no influence on the cell viability of three cells. When the concentrations of PCC-CDs reached 2500 μg/mL, the survival rates of RAW 264.7, LO2 and 293T cells were still up to 80%, suggesting that PCC-CDs exhibited very low cytotoxicity.

### Effects of PCC-CDs on morphological features and Psoriasis Area and Severity Index (PASI) scores

PCC-CDs prepared at 400 ℃ possessed better anti-psoriasis efficacy than that prepared at 325 ℃ and 475 ℃ (Additional file 1: Fig S2). Based on this, the dose-effect relationship of PCC-CDs in the treatment of psoriasis-like inflammation has been further studied.

**Fig 5.**
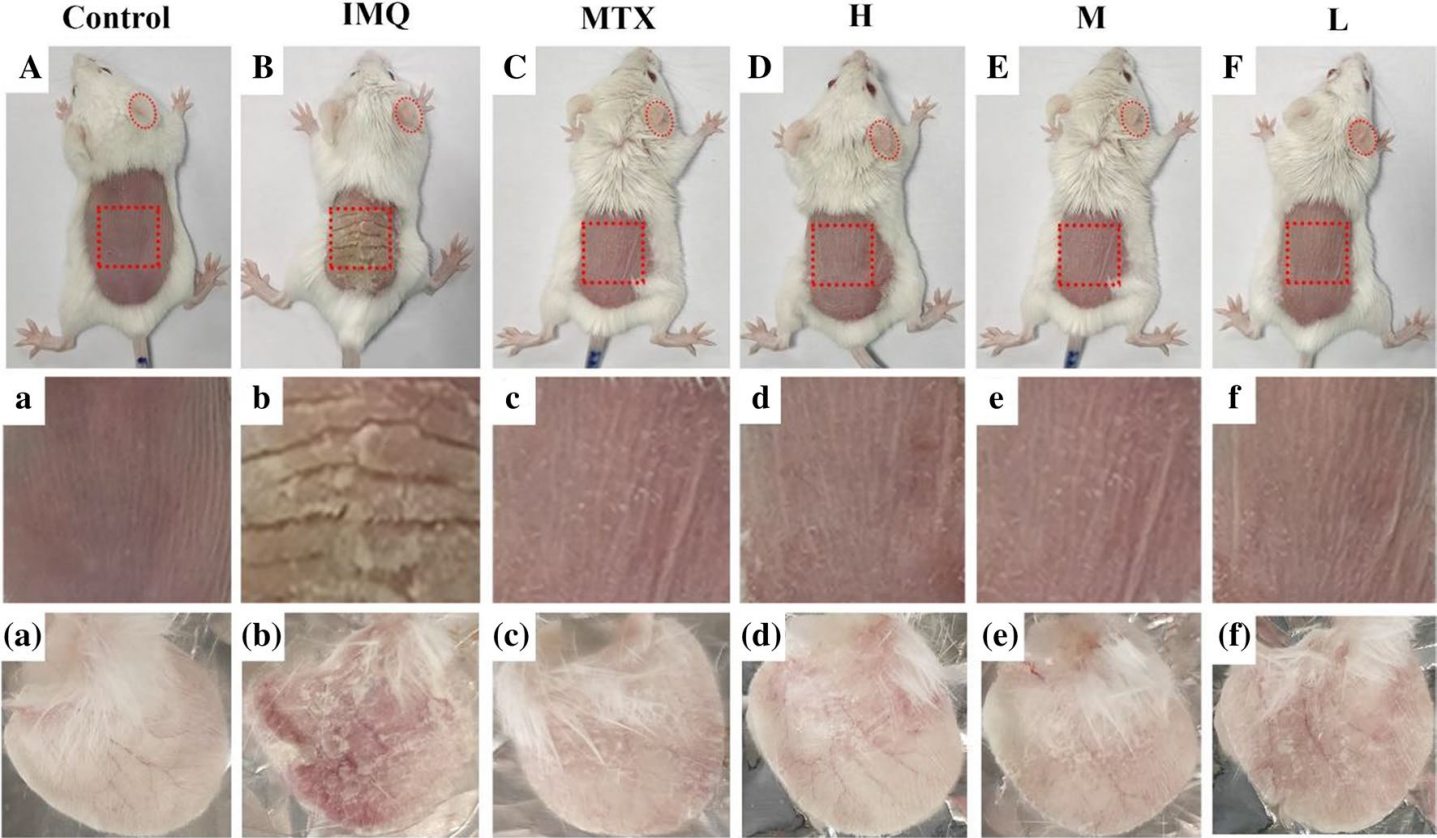
Effects of Phellodendri Chinensis Cortex-based carbon dots (PCC-CDs) on appearance of the dorsal skin and right ears tissues in imiquimod (IMQ)-induced psoriasis-like mice on day 7. **A**–**F** Presentation of phenotype of animal entirety, **a**–**f** Magnification of dorsal skin and (**a**–**f)** right ear tissues. Mouse were assigned into six groups: control (Normal saline [NS], 0.5 mL), IMQ (NS,0.5 mL), methotrexate [MTX] (1.0 mg/kg), different doses of PCC-CDs groups (High[H]: 0.86 mg/kg; Medium[M]: 0.43 mg/kg; Low[L]: 0.22 mg/kg). Except for control group, IMQ and drug-treated group were received a daily application of IMQ

A demonstration of cutaneous and ear psoriasis-like inflammation in different groups have been exhibited in Fig 5. Compared to control group, typical symptoms consisting of erythema, infiltration, thicken skin/ear and scaling could been distinctly observed in IMQ-treated model group. After treatment with methotrexate (MTX) or different doses of PCC-CDs, psoriasis-like appearance of skin and right ear have apparently improved to some extent, indicating the protective effect of PCC-CDs on IMQ-induced psoriasis-inflammation.

**Fig 6.**
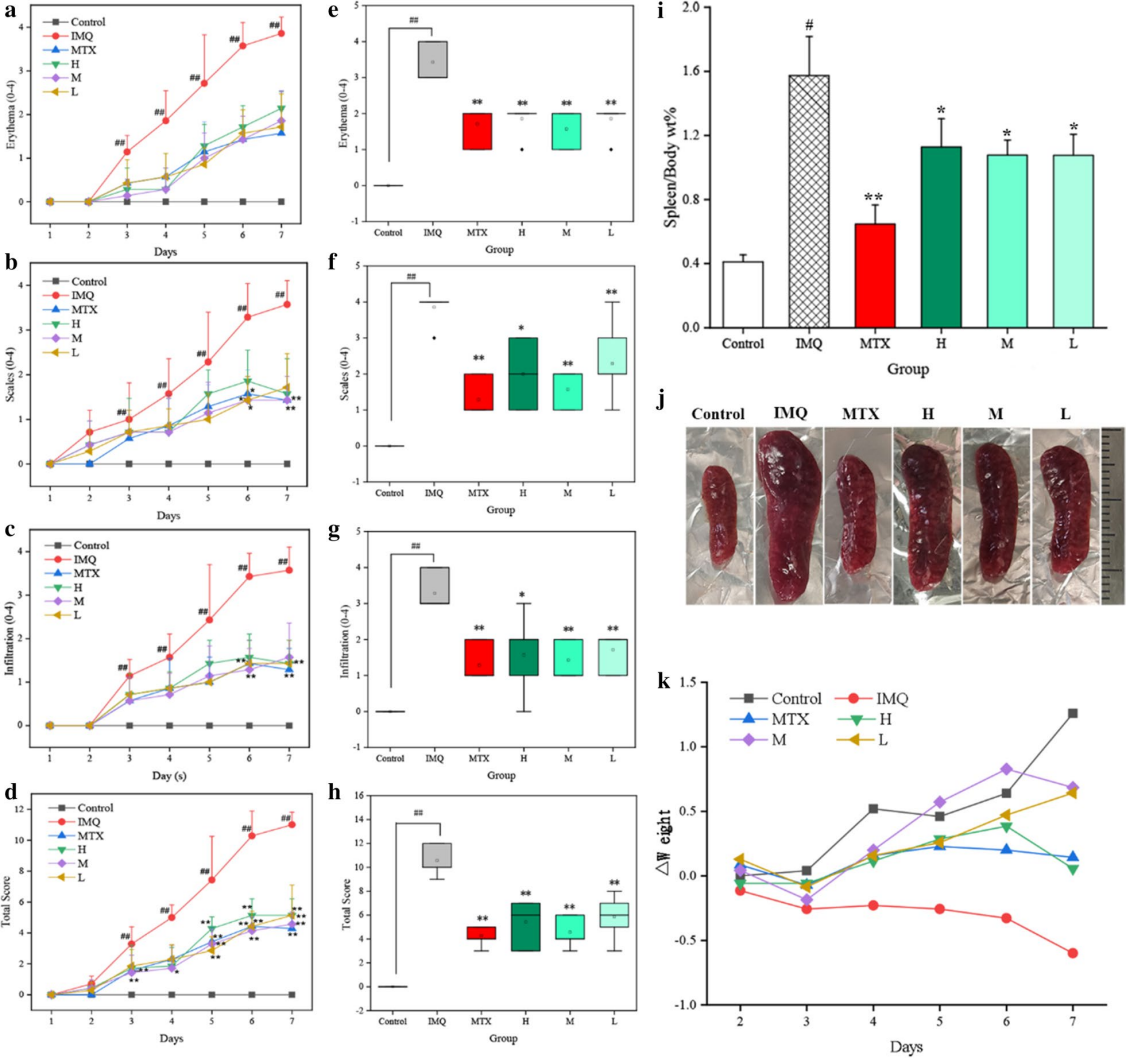
Effects of Phellodendri Chinensis Cortex-based carbon dots (PCC-CDs) on PASI scores (Erythema, scales, infiltration and total scores) of **a**–**d** skin and **e**–**h** right ears, **i** weight ratio of spleen to body [Spleen/body wt%], **j** size of spleen organ and **k** body weight loss (△weight). Mouse were assigned into six groups: control (Normal saline [NS], 0.5 mL), imiquimod [IMQ] (NS, 0.5 mL), methotrexate [MTX] (1.0 mg/kg), different doses of PCC-CDs groups (High[H]: 0.86 mg/kg; Medium[M]: 0.43 mg/kg; Low[L]: 0.22 mg/kg). Except for control group, IMQ and drug-treated group were received a daily application of IMQ. Data were expressed as means ± standard deviation (SD). ^#^*P* < 0.05 and ^##^*P* < 0.01 vs. control group, *P < 0.05 and **P < 0.01 vs. IMQ group

Consistent with the appearance of skin and right ears, PASI scores including erythema, skin/ear thickness, scaling and cumulative scores in IMQ intervention mouse have a gradually increment on consecutive 7 days compared to normal saline (NS)-treated group (P < 0.01), as illuminated in Fig. 6a–h. Typical psoriasis-like symptoms were also developed in all drug-treated groups, but a significant amelioration could be observed in both skin and ears tissues after MTX or PCC-CDs administration. Of note, medium dose CDs-treated group exhibited markedly lower PASI scores than high- and low-dose groups.

### Effects of PCC-CDs on body weight and the weight ratio of spleen to body (Spleen/body wt%)

Furthermore, in contrast to the continued weight loss of mouse in model group on consecutive 7 day, a faint drop in 2 day and gradually augment on days 3 ~ 7 have been observed in high-, medium- and low doses PCC-CDs group. Similar observations were made with regarded to the mouse treated with MTX. In addition, control group showed a continuous increase tendency on body weight. These results suggested PCC-CDs could relieve body weight loss induced by IMQ (Fig. 6k).

It was worthy to note that spleen as the immune organ, the change in its weight and corresponding ratio were linked to immune stimulation or depletion [26, 27]. Spleen/body wt% in model group were approximately 4 times higher than that of NS-treated group (Fig. 6i, P < 0.01), indicating the occurrence of immune activation in IMQ-induced psoriasis-like model. Similar phenomenon was observed in the size of spleen tissues between model and control group (Fig. 6j). After application of PCC-CDs at high-, medium- and low doses for 7 days, all of spleen/body wt% as well as the size of spleen tissue showed a prominent reduction (P < 0.05), and the lowest spleen/body wt% was shown in medium-dose PCC-CDs group. The decreasing trend in spleen/body wt% and the size of spleen tissue were same as MTX-treated group (P < 0.01).

### Effects of PCC-CDs on serum cytokines level

**Fig 7.**
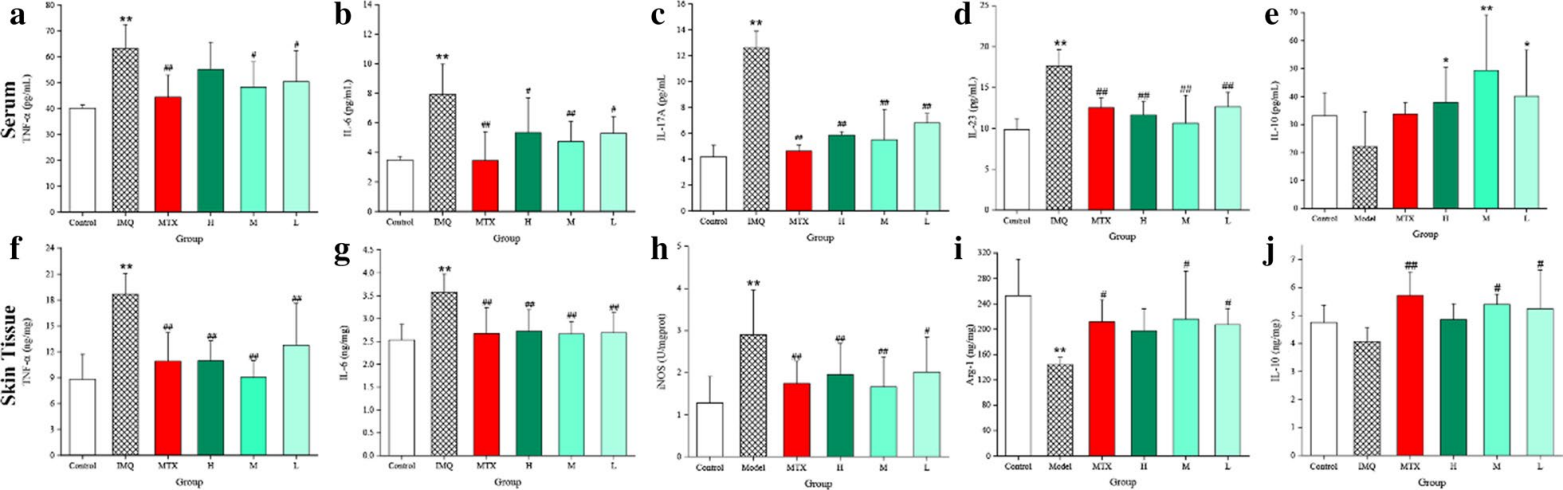
Effects of Phellodendri Chinensis Cortex - based carbon dots (PCC-CDs) on the **a** serum levels of TNF-α, IL-6, IL-17A, IL-23 and IL-10 and **b** skin tissues levels of M1/M2-related cytokines. Control: control group; imiquimod (IMQ): IMQ-treated model group; MTX: methotrexate-treated positive group; H, M, L: high-, medium- and low doses of PCC-CDs group. Data were expressed as means ± standard deviation (SD). *P < 0.05 and **P < 0.01 vs. control group, ^#^*P* < 0.05 and ^##^*P* < 0.01 vs. IMQ group

Figure 7 exhibited the effects of PCC-CDs on the levels of serum cytokines (TNF-α, IL-6, IL-17A, IL-10 and IL-23) by ELISA method. Serum TNF-α (Fig. 7a), IL-6 (Fig. 7b), IL-17A (Fig. 7c) and IL-23 (Fig. 7d) levels were significantly increased in IMQ-treated mouse as compared to control group (P < 0.01), while MTX intervention could relieving the accentuated tendency of four proinflammatory cytokines levels (P < 0.01). In terms of serum level of TNF-α, PCC-CDs at medium and low doses showed significantly decreased relative to model group (P < 0.01). Although high-dose PCC-CDs don’t achieve statistical difference on the serum TNF-α level, a distinct decreasing tendency could be observed in Fig. 7a. Additionally, both serum levels of IL-6 (P < 0.01), IL-17A (P < 0.01) and IL-23 (P < 0.01) were remarkably supressed by medium-dose PCC-CDs, and similarity results could be observed in mouse treated with high- and low doses PCC-CDs (IL-6: P < 0.05 in both doses; IL-17A and IL-23: P < 0.01 in both doses). Serum IL-10 levels in model group was lower than that of the control group. After PCC-CDs intervention, a significant elevation could be observed (H, L: P<0.05 and M: P < 0.01 as compared to model group), which has been shown as Fig. 7e.

### Effects of PCC-CDs on M1/M2-related cytokines levels in skin tissues

A pronounced elevation (P<0.01) of the levels of M1 type inflammation mediators (TNF-α, IL-6 and iNOs) could be observed in IMQ-treated skin (Fig 7f–h) compared with control samples. Of note, treatment with MTX and PCC-CDs at high-, medium- and low doses performed a significant improvement (P < 0.01) on the elevated levels of proinflammation cytokines (TNF-α, IL-6) in skin samples compared to IMQ-treated skin tissues. A similar pattern was also seen for iNOs level in MTX and PCC-CDs-treated mouse (Except PCC-CDs at low dose: P < 0.05).

Additionally, Arg-1 levels (Fig 7i) in skin tissues showed a significant decrease after treating with IMQ (P<0.01 vs. control group), and similar results were found for the IL-10 level (Fig 7j). A significantly upregulated Arg-1 and IL-10 levels, signaturing of M2 macrophages, were been seen in MTX (IL-10: P < 0.01, Arg-1:P<0.05) and medium-, low doses of PCC-CDs-treated (P<0.05 in both indicators) mouse as compared with IMQ mouse. Although there was no significant difference between the high dose PCC-CDs-treated group and IMQ group, an elevated tendency could be observed.

The abovementioned results suggested that PCC-CDs could decreased the elevated M1-related inflammatory mediators induced by IMQ and increased the M2 type anti-inflammation mediators.

### Histopathological analysis

**Fig 8.**
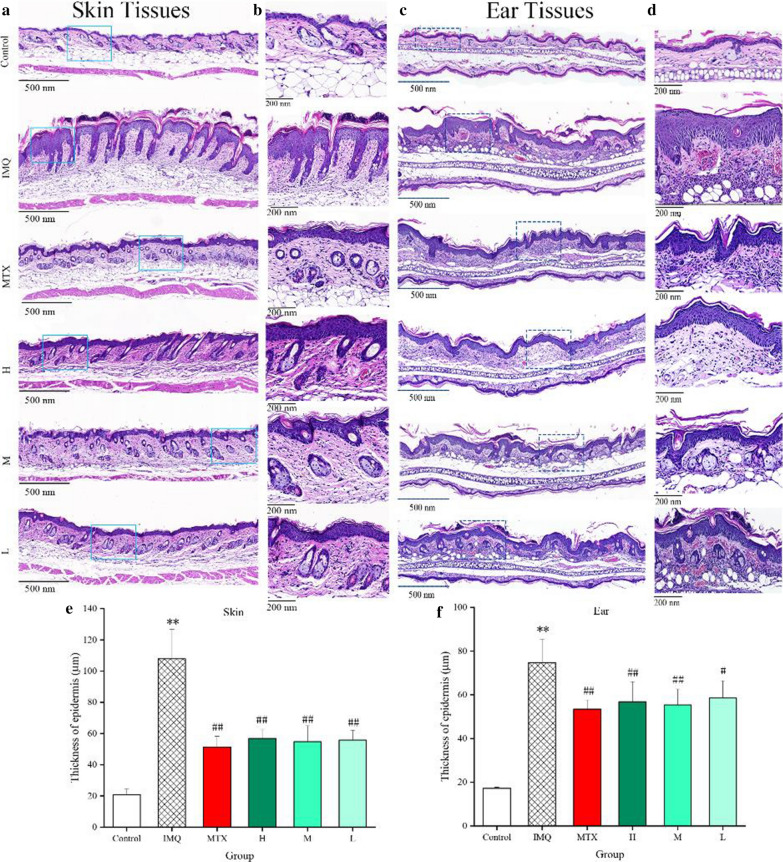
Representative microphotographs and epidermal thickness of **a**, **b**, **e** dorsum skin and **c**, **d**, **f** right ear tissues of mouse. Animals were divided into control, model (only treated with imiquimod [IMQ]), positive (treated with methotrexate [MTX]), Phellodendri Chinensis Cortex-based carbon dots (PCC-CDs) at high[H]-(0.86 mg/kg), medium[M]-(0.43 mg/kg) and low[L] (0.22 mg/kg) doses groups (**a** and **c** H & E staining; magnification×5; **b** and **d** H & E staining; magnification×10). Data were expressed as means ± standard deviation (SD). *P < 0.05 and **P < 0.01 vs. control group,  ^#^P < 0.05 and ^##^P < 0.01 vs. IMQ group

Based on results of histological estimation, typical histopathological changes marked thicker epidermal layers of both skin (Fig 8a, b) and ear (Fig 8c, d) lesions could be observed in IMQ-treated model group, mainly manifesting in severe acanthosis and increasing keratinocytes numbers. These histological observations were accordance with phenotypes common of skins of human psoriasis [28]. MTX and PCC-CDs pre-treatment (0.86, 0.43 and 0.22 mg/kg) led to a prominent alleviation in both dorsal skin and ear tissues consisting of epidermal thickening, less parakeratosis and smoother skin relative to model group. Especially the thickening of epidermal layer in both dorsal skin (Fig. 8e) and ear (Fig. 8f) tissues, data in PCC-CDs or MTX groups were statistical significance (P<0.01 in all group except PCC-CDs at low dose [P < 0.05] in ear tissue) relative to IMQ-induced group.

### PCC-CDs promoted M2 type RAW264.7 cell polarization

**Fig 9.**
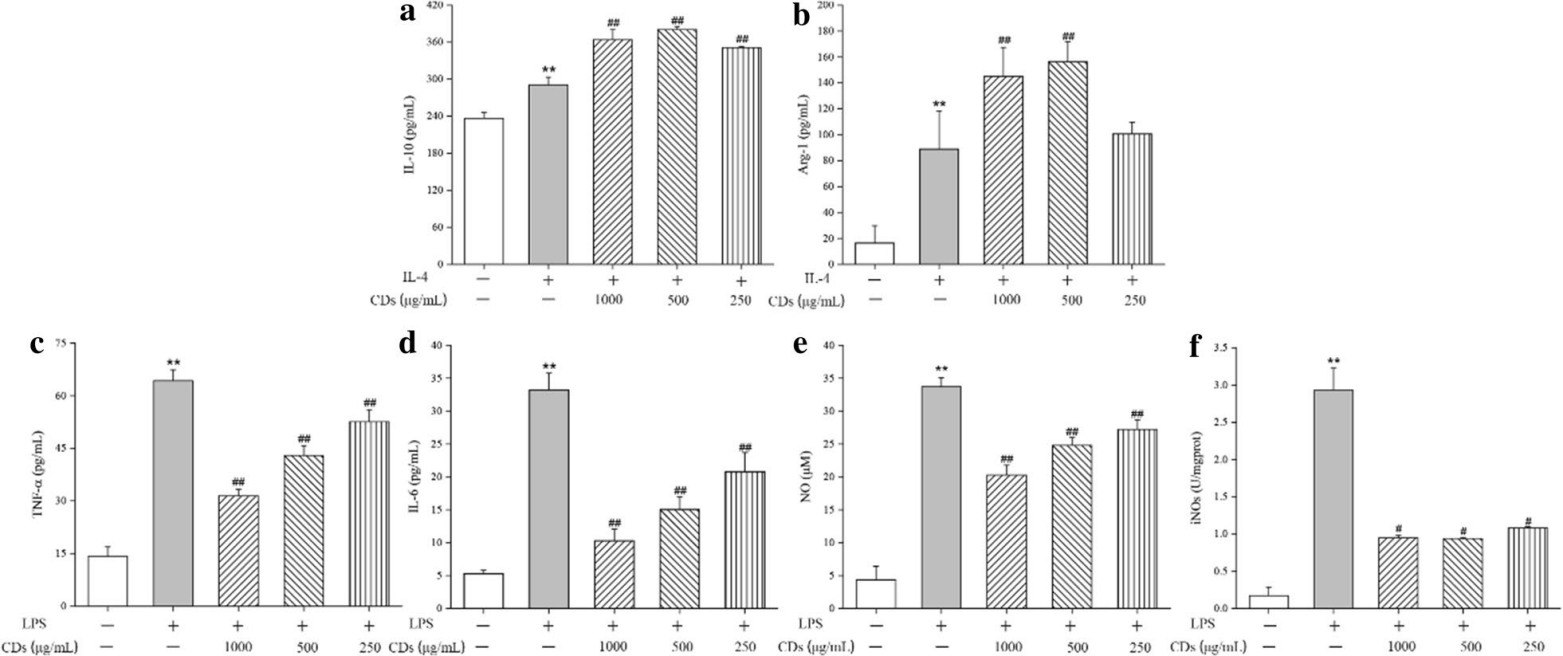
The effects of PCC-CDs on M1/M2 macrophages polarization. The production of **a** IL-10 and **b** Arg-1 in M2 macrophages. The production of **c** TNF-α, **d** IL-6, **e** NO and **f** iNOs in M1 macrophages. *P < 0.05 and **P < 0.01 vs. control group, ^#^P < 0.05 and ^##^P < 0.01 vs. LPS or IL-4 group

The elevated production of M2-associated markers including IL-10 and Arg-1 could be seen in RAW264.7 cells supernatant stimulated by IL-4 compared to that without stimulating by IL-4, indicating that the cells have been successfully polarized into M2 type cells (Fig 9a and b). Notably, PCC-CDs induces a higher expression of M2 type markers in cells than that only incubated with IL-4.

### PCC-CDs inhibited M1 type RAW264.7 cell polarization

The LPS polarized M1 macrophages was examined for the levels of inflammatory mediators (TNF-α, IL-6, NO and iNOs) in cell supernatant or lysate solution. Showed as Fig. 9c–f, higher amounts of TNF-α (P < 0.01), IL-6 (P < 0.01), NO (P < 0.01) and iNOs (P < 0.01) has been produced by M1 macrophages as compared with cells without LPS stimulation, and treatment with PCC-CDs could relieve the elevated levels of abovementioned inflammatory mediators in a dose-dependent manner.

## Discussion

As the carbon-based nanomaterial latest joining the carbon family, CDs received tremendous attentions with the continuous investigation by numerous researchers. The unique advantages prompted scientists focus on the inherent activity of CDs, but related study is still in its infancy. Plentiful underlying bioactivities were not exploring and discovered up to now, which obstructed the further applications of CDs in biology, particularly in vivo. Against this status quo, CDs with novel bioactivity was deserved to be developed to further broaden their application in bioactive fields.

This study, to the best of our knowledge, firstly revealed the pronounced anti-psoriasis effect of novel nontoxic PCC-CDs with sizes of (1.93 ± 0.53) nm prepared via a simple and green calcination method at 400 ℃ for 1 h conditions. As the sole precursor of synthetic CDs, PCC was a kind of Chinese medicine with many advantages of renewability, abundant chemical component, environmental friendliness, and low-price, suggesting that as-prepared PCC-CDs have the possibility of production on a large scale and endowing with enriched functional groups. The assumption was partly demonstrated by FTIR and XPS spectrum, and active group such as carboxyl, amidogen hydroxy could be found through analysis of corresponding data, which may further provide explanation for the prominent anti-psoriasis bioactivity of PCC-CDs.

Moreover, PCC-CDs obtained in this study exhibited difference in size, the ratio of elementary composition, function groups and other aspects as compared to the PCC-derived CDs reported in recent study [17] (prepared at same calcination method but different synthesis condition [350 ℃, 1 h], Additional file 1: Table S1). Remarkable haemostasis [29] and protecting acute kidney injury induced by *Deinagkistrodon acutus* venom [23] efficacies of PCC-CDs prepared at 350 ℃ have been demonstrated, but there was no obvious effect of relieving psoriasis-skin inflammation discovered in our previous study (Additional file 1: Fig S1). These results indicated the PCC-CDs obtained at 400 ℃ was a kind of novel carbon-based nanomaterials with special bioactivity, although the prepared carbon resource was same as the previous reports. Additionally, PCC-CDs prepared at 400 ℃ exhibited better anti-psoriasis activity as compared to CDs obtained at others’ temperature (325 ℃ and 475 ℃). However, the in-depth mechanism related to structure-function relationship between PCC-CDs prepared at different conditions and the corresponding bioactivity remains a challenge. One hypothesis is that multifaceted properties of CDs such as abundant chemical groups, size, solubility played significant role in their biological application [30–32], which will lead to a slight or huge change in terms of physicochemical properties of PCC-CDs prepared at different temperatures linked to a dramatic alteration of the bioactivity of obtained CDs.

Besides, current research referred to nanoparticles as delivery carriers for psoriasis treatment have been gradually developed [33, 34], in sharp contrast to the little information on the application of CDs for treatment of psoriasis. The brand-new found of the anti- psoriasis efficacy of PCC-CDs in this study filled the gap. By using the classic IMQ-induced psoriasis-like skin model, we preliminarily proved the protective effects of PCC-CDs on elevated PASI scores and severe appearance in dorsal skin tissues and right ears of animals treated with IMQ. PCC-CDs at the doses utilized in this study displayed definite improvement on the typical symptoms of erythema, scales, infiltration and cumulative scores, and the same tendency was also shown in the aspects of the size of spleen organ, body weight loss as well as weight ratio of spleen to body. Histopathological observation was further clearly displayed the smoother epidermis, less epidermal thicken and less parakeratosis in the skin and ears of mouse treated with PCC-CDs than that of only treated with IMQ. These results suggested that PCC-CDs possessed noticeable anti-psoriasis efficacy vs. IMQ-induced mouses.

Additionally, the etiology of psoriasis is still complicated and not full elucidated till now, emerging evidence proposed the important role of the recruitment and activation of macrophages in psoriatic skin lesions [35, 36]. Influenced by microenvironment in vivo, macrophages polarized into the classically activated M1 macrophages and the alternatively activated M2 macrophages. In generally, M1 macrophages driven by different stimuli such as LPS and Th1 cytokines (e.g., IFN-γ) plays a role of mediating inflammation and cells/tissue injury via producing inflammation medicators such as TNF-α, IL-6, NO and iNOs. Polarized by Th2 cytokines (e.g., IL-4), M2 macrophages involved in anti-inflammation and repair process in psoriasis by secretion anti-inflammation cytokines (e.g., IL-10, Arg-1) [37]. Thus, regulating the balance of M1/M2 subtypes macrophages, supressing the M1-related inflammation medicators and promoting M2-related anti-inflammation cytokines may be the potential therapeutic targets for psoriasis [38].

From the study on human psoriasis, both classical and alternative activation makers separately secreted by M1 and M2 macrophages could be found in their skins, and a high ratio of M1/M2 subtype emerged in the peripheral blood of patients with severe psoriasis [39]. In the animal model used in this study, the serum in IMQ-treated mouse showed an elevated level of M1-related cytokines (TNF-α, IL-6, IL-23) along with the decrease of the serum IL-10 level, which was accorded with human psoriasis.

Moreover, an obviously improvement in M1-related inflammation medicators (TNF-α, IL-6, NO and iNOs) and suppression in M2-related anti-inflammation cytokines (IL-10 and Arg-1) emerged in psoriasis lesions in the skins of IMQ-treated mouse, which was consistent with previous reports. Especially proinflammation cytokine TNF-α, the primary mediator related to the pathogenesis of psoriasis, which was released by multiple cells such as macrophages, dendritic cells, as well as B and T cells [40]. Patients with psoriasis and animals treated with IMQ were both detected to have a significantly elevated serum TNF-α levels [41, 42], may be related to the facts that TNF-α could induce NF-κB activation followed by promoting the production of other cytokines and upregulation of TNF-α itself via a positive feedback loop. The elevated cytokines levels in serum and skins further led to a systemic inflammation, followed by the development of multiple complications (eg. cardiovascular diseases, arthritis, metabolic syndrome) that may be related to high mortality [41, 43, 44]. Additionally, the elevation of serum IL-17A level in IMQ-mouse indicated Th17 cell activation, which in turn promoted M1 polarization and suppressed M2 activation.

Based on this, we measured the M1/M2 related cytokines in serum and skin tissues of all animals and the positive effects of PCC-CDs at high-, medium- and low doses have been demonstrated relative to IMQ-induced animals, suggesting the inflammatory status have been relieved satisfactorily after the administration of as-prepared CDs via suppressing M1 activation and relatively promoting M2 polarization. The cell experiment results further verify this conclusion.

Thus, the present study, for the first time, exhibited novel perspective in the pronounced bioactivity of non-toxic PCC-CDs for anti-psoriasis efficacy by regulating of M1/M2 polarization, providing potential hope for the development of application nanodrugs (eg. CDs) to treat complicated and incurable diseases. Additionally, research on the in-depth mechanism should be conducted in further study.

## Conclusion

Novel and non-toxic CDs derived from PCC were synthesized by means of one-step calcination methods without addition of any organic reagent. The as-prepared PCC-CDs with tiny size and abundant chemical groups exhibited prominent anti-psoriasis function in IMQ-induced inflammation skin model, preliminary demonstrated to be related to regulating of M1/M2 macrophage polarization. The result in this work holds a promising potential for PCC-CDs to be a viable alternative to current strategies for psoriasis treatment.

## Materials and methods

### Materials

PCC was purchased from Beijing Qiancao Herbal Pieces Co., Ltd (Beijing, China). Dialysis membranes (1000 MWCO) were obtained from Beijing Ruida Henghui Technology Development Co., Ltd (Beijing, China). MTX was obtained from Beijing BioDee Biotechnology Co, Ltd (Beijing, China). Imiquimod cream was obtained from Sichuan Mingxin Pharmaceutical Co., Ltd. (Sichuan, China). Mouse TNF-α, IL-6, IL-17A, IL-23, IL-10 and Arg-1 ELISA kits were brought from Proteintech Group, Inc. (Chicago, USA). INOs Biochemical kit was purchased from Nanjin Jiancheng Bioengineering Institute (Nanjin, China).The cell counting kit-8 was procured from Dojindo Molecular Technologies, Inc. (Kumamoto, Japan). Lipopolysaccharide (LPS) and Recombinant Mouse IL-4 was brought from sigma-aldrich, Inc. (shanghai, China) and Novoprotein Scientific Inc. (Shanghai, China). Furthermore, Roswell Park Memorial Institute (RPMI) 1640 medium, Dulbecco’s modified Eagle’s medium (DMEM), fetal bovine serum (FBS) and 0.25% trypsin-EDTA were purchased from Corning Co., Ltd. (New York, USA). Chemical reagents used throughout this study were from Sinopharm Chemical Reagents (Beijing, China).

### Animals

Male BALB/c mice (22 ~ 25g) purchased from Beijing Vital River Laboratory Animal Technology Co., Ltd (Beijing, China) were housed in a laboratory environment with constant temperature (24.0 ± 1.0°C) and relative humidity (45.0–55.0%) on a 12 h light/dark cycles. These animals had been provided free access to unlimited amounts of food and water during the entire experiment.

### Preparation of PCC-CDs

Green CDs derived from PCC were prepared by a one-step calcination method (Fig. 1), as previously reported [45]. Briefly, PCC placed in crucible was heated in a pre-heated furnace at 325 ℃, 400 ℃ and 475 ℃ for 1 h, respectively, followed by grounding into fine powder after the furnace cooling to 40 ℃. The black power was dispersed in deionized water and boiled twice at 100 ℃. Then 0.22 μm microporous membrane was employed to remove the larger products. The CDs were collected followed by dialysis against deionized water through a dialysis bag (1000 MWCO) for 1 week and stored at 4 °C for further application.

### Characterization

The morphology and structural details of PCC-CDs were observed by a JEM-2100 transmission electron microscope (JEOL, Tokyo, Japan) and a Tecnai G2 20 high-resolution TEM (HRTEM; JEOL, Tokyo, Japan). Fluorescence and absorption properties were obtained on fluorescence spectroscopy (F-4500, Tokyo, Japan) and UV–Vis spectrophotometry spectroscopy (CECIL, Cambridge, UK), respectively. The functional group properties of PCC-CDs prepared at 400 ℃ were recorded on a Fourier transform infrared spectroscopy spectrophotometer (Thermo Fisher Scientific, CA, USA). X-ray photoelectron spectroscopy (ESCALAB 250Xi, Thermo Fisher Scientific, CA, USA) was applied to acquire the chemical groups and elemental composition information of PCC-CDs prepared at 400 ℃.

### Fluorescence property of PCC-CDs

Studies on fluorescence properties of PCC-CDs prepared at 400 ℃ in aqueous solution were mainly composed of two parts: the maximum excitation/emission wavelength and QY, which were carried out on a fluorescence spectroscopy.

Besides, the QY of PCC-CDs was measured according previous method using quinine sulfate (0.1 M H_2_SO_4_ aqueous solution, QY = 0.54) as reference [46]. The following equation was used to calculate the absolute values.

$${\text{Q}}_{\text{CDs}}{=}{\text{Q}}_{\text{R}}\times\frac{{\text{I}}_{\text{CDs}}}{{\text{I}}_{\text{R}}}\times\frac{{\text{A}}_{\text{R}}}{{\text{A}}_{\text{CDs}}}\times\frac{{\eta}_{\text{CDs}}^{2}}{{\eta}_{\text{R}}^{2}}$$

where Q is the QY, Ι represents the measured integrated emission intensity obtained at 330 nm, A is the absorbance at 330 nm wavelength, and η is the refractive index of the solvent. The subscript "R" and "CDs" refers to standard and PCC-CDs, respectively. For aqueous solution, η_CDs_/η_R_ = 1.The R and CDs with optical absorbance less than 0.05 were prepared to minimize the reabsorption interference.

### Cytotoxicity assay

In vitro cytotoxicity of obtained PCC-CDs prepared at 400 ℃ was evaluated by a standard CCK-8 assay. RAW 264.7 mouse macrophage, Human L02 hepatocyte and embryonic kidney 293T cell lines were seeded at 2 × 10^4^ cells/well in a 96-well plate and incubated for 24 h under a 37 ℃ and 5% CO_2_ atmosphere, respectively. After treated with different concentrations of PCC-CDs (2500, 1250, 625, 313, 156, 78, 39 μg/mL) for 24 h (following by washing twice with PBS), 10 μL CCK-8 was added and incubated for another 2 h. Additionally, the control cells were treated with above media, as appropriate. The absorbance of each well was recorded at 450 nm by a microplate reader (Biotek, USA). Cell viability (%) was calculated according to following formula using serum-free RPMI 1640 or DMEM as the blank.

$${Cell \,viability \,(\%\, of\,  control) =}\frac{\text{Aa}-\text{Ac}}{\text{Ab}-\text{Ac}} \times 100$$

Aa, Ab, and Ac represent the absorbance of the experimental, control, and blank groups at 450 nm, respectively.

### The anti-psoriatic activity of PCC-CDs prepared at different temperatures

The optimizing preparing conditions of PCC-CDs with anti-psoriatic activity was assessed in this study. Animals were assigned into six groups of 7 mice per group: control group (NS, 0.5 mL, i.p.), model (NS, 0.5 mL, i.p.), positive (MTX, 1.0 mg/kg, i.p.) and PCC-CDs at different preparing temperature conditions (325 ℃, 400 ℃ and 475 ℃, i.p.). The administration dosage of PCC-CDs obtained at three conditions were 0.22 mg/kg. Animals in model and drug treatment groups, but not in control group, received a daily application of 62.5 mg of 5% IMQ to a shaved epidermal area on their back for 7 consecutive days to induce psoriasis-like inflammation skin [47].

To further monitor and grade the severity of the psoriasis-inflammation skin condition of PCC-CDs prepared under different conditions, cumulative scores of on day 8 were employed as previous reported [28]. The parameter was separately scored from 0 to 4 (0: none; 1: mild; 2: moderate; 3: severe; 4: very severe), while cumulative scores represented the final indication of the inflammation severity on day 7.

These animals were euthanized on the eighth day and the back skin samples were preserved in a 4% paraformaldehyde solution, followed by embedded in paraffin. For histopathological examination, 4 µm sections were stained with hematoxylin and eosin (H&E) and observed by a light microscope.

### In vivo anti-psoriatic activity of PCC-CDs obtained at 400 ℃

The mouse were randomly distributed into 6 groups of 7 mice per group: control group (NS, 0.5 mL, i.p.), model (NS, 0.5mL, i.p.), positive (MTX, 1.0 mg/kg, i.p.) and PCC-CDs at different doses (high[H]: 0.86 mg/kg, medium[M]: 0.43 mg/kg, low[L]: 0.22 mg/kg; i.p.). Except for control groups, animals in all other groups were received a daily application of 62.5 mg of 5% IMQ to a shaved epidermal area on their back and right ear for 7 consecutive days to induce psoriasis-like inflammation skin and ear. Body weight and PASI Scores (consisting of skin erythema, scaling and infiltration and cumulative scores, detailed evaluation criteria shown as Additional file 1: Table S2) of all animals were recorded daily.

These animals were euthanized on the eighth day and the spleen was harvested and weighted, followed by calculating spleen/body wt%. In addition, blood and skin tissues were immediately collected for histological and M1/M2 related cytokines analysis.

The serum collected by centrifuging blood at 3000 rpm for 10 min at 4 ℃ was used for measuring the levels of TNF-α, IL-6, IL-23, IL-17A and IL-10 using commercially ELISA kits. Skin tissues from different groups were weighed and homogenized with PBS on ice, followed by centrifuged at 6000 rpm for 15 min. The levels of TNF-α, IL-6, IL-10, Arg-1 in tissue supernatant was measured by ELISA kits and the iNOs level was determined by biochemical kits. Absorbance at 450 nm and 540 nm (iNOs) was recorded with a microplate reader, respectively.

The others’ back skin and right ear samples were preserved in a 4% paraformaldehyde solution, followed by embedded in paraffin. For histopathological examination, 4 µm sections were stained with hematoxylin and eosin (H&E) and observed by a light microscope. Epidermal thickness of both skin and right ear tissues were separately obtained by measuring the average thickness of 15–20 random fields, blinded to treatment group.

### M1/M2 type cell polarization and PCC-CDs administration

RAW 264.7 cells were seeded at 8 × 10^4^ cells/well in a 24-well plate and incubated for 12 h. After treating with different concentrations of PCC-CDs (1 mg/mL, 0.5 mg/mL and 0.25 mg/mL) or free DMEM medium for 2 h, cells were polarized to M1 or M2 macrophages by using 1 μg/mL LPS or 20 ng/mL IL-4 for 24 h, respectively. Then the level of cytokines (TNF-α, IL-6, IL-10 and Arg-1) in culture media was determined by enzyme-linked immunosorbent method. The iNOs level of M1 macrophages was measured by biochemical analysis method.

### Statistical analysis

All data analyses were performed using IBM SPSS statistics software (version 20). Results were shown as means ± standard deviation (SD). Differences between groups were performed by one-way ANOVA followed by the least significant difference test or Dunnett’s T3 method. *P* value < 0.05 was considered statistically significant and *P* value < 0.01 was most significant.

## Supplementary Information

**Additional file 1: Table S1.** The comparative analysis of the data between CDs derived from PCC at different temperature (350 ℃ and 400 ℃) by calcination method. **Table S2.** PASI scoring criteria in imiquimod-induced psoriasis-like inflammation mouse. **Figure S1.** The detailed description about the effects of PCC-CDs prepared at 350 ℃ for 1 h by calcination method on appearance of the dorsal skin in imiquimod (IMQ)-induced psoriasis-like mice on day 1, 3, 7. **Figure S2.** The comparison of anti-psoriasis activity among PCC-CDs prepared at 325 ℃, 400 ℃ and 475 ℃.

## Data Availability

Data sharing is not applicable to this article as no datasets were generated or analysed during the current study.

## Abbreviations

CDs: Carbon dots
PCC: Phellodendri Chinensis Cortex
IMQ: Imiquimod
PCC-CDs: PCC-based CDs
CCK-8: Cell counting kit-8
TEM: Transmission electron microscope
UV–Vis: Ultraviolet and visible
QY: Quantum yield
FTIR: Fourier transform infrared spectroscopy spectrophotometer
XPS: X-ray photoelectron spectroscopy
PASI: Psoriasis Area and Severity Index
MTX: Methotrexate
NS: Normal saline
LPS: Lipopolysaccharide
