# Supplementary material for: Green Phellodendri Chinensis Cortex-based carbon dots for ameliorating imiquimod-induced psoriasis-like inflammation in mice
Source: J Nanobiotechnology. 2021 Apr 15;19:105. doi: 10.1186/s12951-021-00847-y (PMC8048166; doi:10.1186/s12951-021-00847-y)
Supplement: Supplementary file 1 — Additional file 1: Table S1. The comparative analysis of the data between CDs derived from PCC at different temperature (350 ℃ and 400 ℃) by calcination method. Table S2. PASI scoring criteria in imiquimod-induced psoriasis-like inflammation mouse. Figure S1. The detailed description about the effects of PCC-CDs prepared at 350 ℃ for 1 h by calcination method on appearance of the dorsal skin in imiquimod (IMQ)-induced psoriasis-like mice on day 1, 3, 7. Figure S2. The comparison of anti-psoriasis activity among PCC-CDs prepared at 325 ℃, 400 ℃ and 475 ℃. [file 12951_2021_847_MOESM1_ESM.pdf]

Additional file

Green Phellodendri Chinensis Cortex - Based Cardon Dots for Ameliorating Imiquimod-Induced Psoriasis-like inflammation in Mice

Meiling Zhang[1#], Jinjun Cheng[1#], Jie Hu [1], Juan Luo [1], Yue Zhang[2], Fang Lu[2], Hui Kong[1*], Huihua Qu[3*], and Yan Zhao [1*]

[1]*School of Traditional Chinese Medicine, Beijing University of Chinese Medicine, Beijing 100029, China;*

[2]*School of Life Sciences, Beijing University of Chinese Medicine, Beijing 100029, China;*

[3]*Center of Scientific Experiment, Beijing University of Chinese Medicine, Beijing 100029, China*

Table S1. The comparative analysis of the data between CDs derived from PCC at different temperature (350 °C and 400 °C) by calcination method

Characterization	data	
	350℃	400℃
Particle size distribution (nm)	2.84 ± 0.89	1.93 ± 0.53
lattice structure (nm)	0.24	0.22
ultraviolet absorption(nm)	265	270 and 350
Maximum excitation and emission wavelength (nm)	370,445	330,445
Functional groups	O–H/N–H, C–H, C=O, and C–O	O–H/N–H, C–H, C=O, and C-O
Element analysis	C: 71.64%<br>O: 23.26%<br>N: 1.30%	C: 67.61%<br>O:24.19%<br>N:3.81%
Bioactivity	Hemostasis, protecting kidney injury induced by Deinagkistrodon acutus venom	Anti-psoriasis

Table S2. PASI scoring criteria in imiquimod-induced psoriasis-like inflammation mouse

Scores	Severity	Erythema	Scales	Infiltration
0	None	None	None	None
1	Mild	Mild red	Local skin lesions with minor scales	Mild bulge
2	Moderate	Red	About half of the skin lesions with flaky scales	Moderate bulge
3	Severe	Dark red	Most skin lesions with thicker flaky scales	Severe bulge
4	Very severe	Purplish red	All skin lesions with layers of scales	Very severe bulge

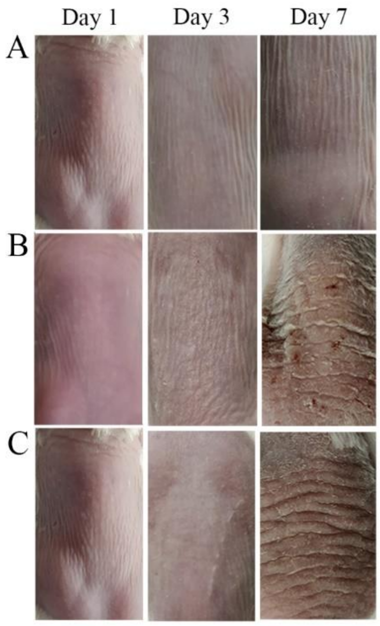

Fig S1. The detailed description about the effects PCC-CDs prepared at 350 °C for 1 h by calcination method on appearance of the dorsal skin in imiquimod (IMQ) - induced psoriasis-like mice on day 1,3,7.

Animals in IMQ-treated group distinctly showed erythema, infiltration and scaling symptoms, indicating the successfully establishing of psoriasis-like inflammation skin model. After administration of PCC-CDs prepared by the method as previous reported, the appearance of dorsal skin was nearly same as the IMQ-treated group (Fig S1). The above-mentioned result was in contrast to the significant anti-psoriasis effect of PCC-CDs prepared in this study, which suggested that the difference in physicochemical properties of PCC-CDs synthesized at different temperature led to an alteration of the bioactivity.

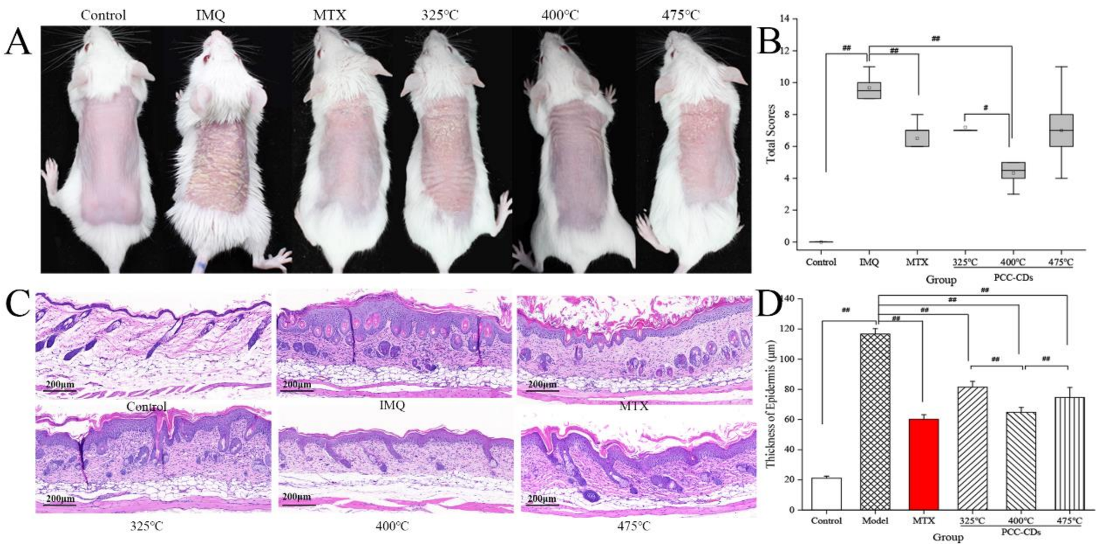

Fig S2. The comparison of anti-psoriatic activity among PCC-CDs prepared 325°C, 400°C and 475°C.

The anti-psoriasis activity of PCC-CDs prepared at different temperature (325 °C, 400 °C and 475 °C) has been studied by IMQ-induced psoriasis - like inflammation skin model. Shown as figure S2 (A-D), dramatic changes in the clinical symptoms of the dorsal skin, PASI total scores, epithelial thickness and pathological morphology could be observed in model group. After PCC-CDs obtained at 400 °C intervention, the mouse performed a significant amelioration in the aspects of clinical symptoms including erythema, infiltration, scales as well as total scores ($P < 0.01$ as compared to model group). Similar observations were made with regard to the pathological changes and epithelial thickness ($P < 0.01$ vs. model group).

Additionally, mouse treated with PCC-CDs prepared at 325 °C and 475 °C displayed an improvement in the appearance of the dorsal skin, but the PASI total scores showed no statistic difference as compared to model mouse. In terms of pathological changes, both PCC-CDs prepared at 325 °C ($P < 0.01$) and 475 °C showed a smoother epidermis, reduced epidermal thickening ($P < 0.01$ vs. model group) and less parakeratosis, indicating that the afore-mentioned PCC-CDs performed certain anti-psoriasis activity.

Of note, mouse treated with PCC-CDs obtained at 400 °Cexhibited lower total scores ($P < 0.05$ vs. PCC-CDs obtained at 325 °C) and minor pathological changes such

as epidermal thickening ($P < 0.01$ vs. PCC-CDs obtained at 325 ℃ and 475 ℃), parakeratosis than that of in PCC-CDs obtained at 325 ℃ and 475 ℃ group. These results suggested that the preparation temperature of PCC-CDs has a great influence on its activity in the treatment of psoriasis and PCC-CDs obtained at 400 ℃ displayed an optimum anti-psoriasis activity.
